# The protective effect of boric acid on cholestatic rat liver ischemia reperfusion injury

**DOI:** 10.3906/sag-2101-153

**Published:** 2021-10-21

**Authors:** Serkan GÜLER, Arif ASLANER, Hamit Yaşar ELLİDAĞ, Şenay YILDIRIM, Tuğrul ÇAKIR

**Affiliations:** 1 Department of General Surgery, Antalya Training and Research Hospital, University of Health Sciences, Antalya Turkey; 2 Department of Biochemistry, Antalya Training and Research Hospital, University of Health Sciences, Antalya Turkey; 3 Department of Pathology, Antalya Training and Research Hospital, University of Health Sciences, Antalya Turkey

**Keywords:** Boric acid, liver, cholestasis, ischemia reperfusion

## Abstract

**Background/aim:**

To evaluate the potential protective effects of boric acid (BA) in experimental cholestatic liver ischemia reperfusion (IR) injury model.

**Materials and methods:**

The study included 24 female rats which were divided into 3 groups each containing 8 rats. The control group (Group 1) only received laparotomy. In the IR group (Group 2) biliary tract ligation was applied and 1 week later 45 min ischemia and 1 h reperfusion with relaparotomy without any treatment was implemented. In the treatment BA+IR group (Group 3). 1 week after the biliary ligation intraperitoneal administration of 200 mg/kg BA was given 10 min before the ischemia for 45 min and reperfusion for 1 h with relaparotomy. Liver tissue and blood samples were taken for histopathological and biochemical examination. Ischemia modified albumin (IMA), SCUBE1, total antioxidant status (TAS), and total oxidant status (TOS) levels were also examined.

**Results:**

Compared to control, groups IR and BA+IR had higher serum alanine aminotransferase (ALT), aspartate aminotransferase (AST), alkaline phosphatase (ALP), lactate dehydrogenase (LDH), total, and direct bilirubin levels. Albumin value was high in the control group and low in the other groups. In terms of IMA levels there was no significant difference between groups (p > 0.05). When SCUBE-1 levels were examined groups IR and BA+IR were significantly higher than the group 1. TAS was highest in the group BA+IR whereas TOS was highest in the group IR and lower in the group BA+IR. In histopathological analysis, loss of intercellular border loss in hepatocytes, diffuse nuclear pycnosis and mild to moderate neutrophilic cell infiltration were observed in the IR group. Statistically significant dissociation, hemorrhage and severe neutrophilic cell infiltration were seen in hepatocytes of rats with IR (p < 0.05).

**Conclusion:**

BA has promising results in the treatment of experimental IR injury of the cholestatic liver because of its antioxidant effects. It may be used in clinical practice after more extensive studies about the effects of BA on IR injury of the cholestatic liver.

## 1. Introduction

Ischemia is defined as the interruption or reduction of blood flow to the tissue for any reason. Reperfusion is defined as the normalization of blood flow in ischemic tissue. IR injury is the damage caused by free oxygen radicals (FOR) caused by reperfusion and reperfusion in order to maintain the viability of the tissue [1].

Trauma, shock, liver surgery and transplantation are among the most common causes of liver IR injury in the clinic [2]. Many mechanisms have been described regarding the mechanism of IR injury. As a result of the change in mitochondrial oxidative phosphorylation decrease in ATP increase in intracellular calcium and activation of proteases and phosphatases leading to the deterioration of cytoskeleton and membrane phospholipids excessive FOR is formed and causes oxidative stress [3,4].

In the presence of the liver is more susceptible to reperfusion damage [5,6]. When bile flow is interrupted sinusoidal endothelial cell damage accelerates; neutrophil accumulation and kuppfer cell activation are stimulated [6]. Activated neutrophils and kuppfer cells are the most important sources of reactive oxygen radicals in oxidative stress [7,8]. Cholestasis is a common finding in liver and pancreatic diseases requiring major hepatic surgery; cholestatic IR models are more valuable to reflect this situation.

cholestasisthereforeIMA is an albumin that changes its structure as a result of ischemia reperfusion injury. In addition to ischemia, IMA may increase in acute coronary syndrome, cerebrovascular diseases, liver failure, end-stage renal disease, severe traumas, pulmonary embolism, sepsis, diabetes, systemic sclerosis, some neoplastic diseases and infections. In this way, we believe IMA is more clinically valuable. 

Boron or boric acid (BA) is an element found in nature and is abundant in Turkey. Studies on its clinical use are quite common. BA has an inhibitory effect on peptidases, xanthine oxidase, nitric acid synthase, cytochrome b reductase. In addition it also affects calcium, phosphorus, magnesium ion levels and vitamin D, insulin, testosterone, estrogen and glucose metabolism [9,10]. It has been shown in many studies to have antioxidant and antiinflammatory effects [1114].

--The aim of this experimental study was to investigate the effects of BA on experimental IR injury of the cholestatic liver through the evaluation of oxidative stress and histopathological changes. To the best of our knowledge this is the first study in the literature to have examined the effect of BA on cholestatic liver damage caused by IR. 

## 2. Materials and methods

The approval for this study was granted by the Animal Research Ethics Committee of Antalya Akdeniz University dated 15.01.2019 and numbered B.30.2.AKD.0.05.07.00/7. All procedures were applied at Akdeniz University Experimental Medicine and Animal Laboratory in accordance with the principles of the National Guidelines for Experimental Use of Laboratory Animals between January and March 2019. 

### 2.1. Animals 

In this study 24 female Wistar Albino rats weighing between 200250 were used. During both preoperative and postoperative the rats were housed at cages with constant environmental conditions (temperature: 23°C and humidity: 55.5%) and fed with standard laboratory feed and tap water. Access to food was stopped 12 before anesthesia and to water 2 h prior to anesthesia.

-grams periodshour our2.2. Surgery and experimental protocol 

This research aimed to investigate the effects of BA on IR injury of the cholestatic liver. The rats were randomized into 3 groups;

Group 1 (Control): Only laparotomy was performed. After waiting for 1 and 45 minhour blood and tissue samples were taken and the animals were sacrificed. No other treatment was given. 

Group 2 (IR): After laparotomy the common bile duct was dissected from adjacent tissue and ligated with 3/0 silk sutures. After the procedures each abdominal incision was closed with 3/0 prolene sutures. Subsequently the rats were allowed to feed. On the postoperative 7th day relaparotomy was performed. The portal vein and hepatic artery were clamped and ischemia was performed for 45 min. Reperfusion was performed for 1 h by opening the clamp. No other intervention or medication was given. Liver tissue samples and blood samples from the aorta utesutesourwere taken for histopathological and biochemical analyses and the rats were sacrificed. 

Group 3 (BA+IR): After laparotomy the common bile duct was found and dissected from adjacent tissues and ligated with 3/0 silk sutures. After the procedures each abdominal incision was closed with 3/0 prolene sutures. Subsequently the rats were allowed to feed. On the postoperative 7th day 200mg/kg BA was given intraperitoneally. After waiting for 10 min relaparatomy was performed. The portal vein and hepatic artery were clamped and ischemia was performed for 45 min. Reperfusion was performed for 1 by opening the clamp. Liver tissue sample and blood samples from the aorta were taken for histopathological and biochemical analyses and the rats were sacrificed.

utesuteshour 2.3. Biochemical analysis 

Routine parameters

Rat serum albumin (Beckman Coulter Diagnostics, Brea, CA, USA; catalogue no. OSR6602), alanine aminotransferase (ALT) (Beckman Coulter Diagnostics, Brea, CA, USA; catalogue no. OSR6607), aspartate aminotransferase (AST) (Beckman Coulter Diagnostics, Brea, CA, USA; catalogue no.OSR6509), alkaline phosphatase (ALP) (Beckman Coulter Diagnostics, Brea, CA, USA; catalogue no.OSR6504) total bilirubin (TBIL) (Beckman Coulter Diagnostics, Brea, CA, USA; catalogue no.OSR6512) and (DBIL) (Beckman Coulter Diagnostics, Brea, CA, USA; catalogue no. OSR6511) levels were determined using an autoanalyzer (Beckman AU5800; Beckman Coulter Diagnostics, Brea, CA, USA). 

Direct Bilirubin 2.4. Measurement of SCUBE1

**:**Rat serum SCUBE1 levels were quantified using a commercially available ELISA kit (Bioassay Technology Laboratory, Shanghai, China; catalogue no. E0948Ra) according to the manufacturer’ instructions. The sensitivity of the assay was 0.35 ng/mL with inter- and intra-assay coefficients of variation of <10%. Assay results were expressed as ng/mL.

### 2.5. Measurement of ischemia modified albumin (IMA)

 Reduced cobalt–albumin-binding capacity (IMA level) was measured using the rapid and colorimetric method developed by Bar-Or et al [15]. Briefly 200 rat serum was transferred into glass tubes and 50 0.1% CoClμl μl _2_ * 6H_2_O (lot S38901-248. Sigma Aldrich, St Louis. MO. USA) was added. After gentle shaking the mixture incubated for 10 minto ensure sufficient cobalt–albumin binding. Then 50 1.5 mg/dithiothreitol (DTT) (lot D5545-1G. Sigma-Aldrich) was added as a coloring agent. After 2 minutes μl ml 1 0.9% NaCl was added to halt the binding between cobalt and albumin. A blank was prepared for every specimen: at the DTT addition step. utes.ml 50 μl distilled water was used instead of 50 1.5 mg/ml DTT μl to obtain a blank without DTT. The were recorded at 470 nm with a spectrophotometer (UV1201. Shimadzu, Kyoto Japan). Color formation in specimens with DTT was compared with color formation in the blank tubes and the results are expressed as absorbance units.

absorbances.2.6. Measurement of serum total oxidant status (TOS)

**: **Serum TOS levels were analyzed using an automated colorimetric measurement method developed by Erel [16]. In this method oxidants in the sample oxidize the ferrous ion-chelated complex to ferric ion, which makes a colored complex with a chromogenic in an acidic medium. The color intensity which can be measured spectrophotometry is related to the total amount of oxidant molecules present in the sample. The results are expressed in terms of micro molar hydrogen peroxide equivalent per liter (μmol H_2_O_2 _equiv./l).

### 2.7. Measurement of serum total antioxidant status (TAS)

**: **Serum TAS levels were analyzed using an automated colorimetric measurement method developed by Erel [17]. In this method antioxidants in the sample reduce dark bluegreen colored 22–.′-azino-bis (3-ethylbenzthiazoline-6-sulfonic acid) (ABTS) radicals to a colorless reduced ABTS form. The change of absorbance at 660 nm is related to the total antioxidant level in the sample. This method determines the anti oxidative effect of the sample against the potent free radical reactions initiated by the produced hydroxyl radical. The results are expressed as micro molar Trolox equivalent per liter (μmol Trolox equiv./l). 

-2.8. Oxidative stress index (OSI)

**: **The percentage ratio of the TOS level to the TAS level is given as the OSI [18]. For calculation the resulting micro molar unit of TAS was changed to and the OSI value was calculated according to the following formula: OSI (arbitrary unit) = TOS (μmol Hmillimoles per liter_2_O_2_ equiv./l)/TAS (μmol Trolox equiv./l). 

### 2.9. Histopathological evaluation 

Liver samples were fixed in 10% neutral formalin solution. After 48 of fixation tissues were dehydrated through a series of graded alcohol embedded in paraffin and cut into4μm sections using a microtome (Leica RM 2125. Leica Microsystems Nussloch GmbH. Germany) and stained with hematoxylin and eosin (H&E). These preparations were blindly evaluated by the pathologist under a light microscope.

hours Hepatic injury was evaluated for severity of hepatic injury using an ordinal scale as follows: Grade 0 = Minimal or no evidence of injury; Grade 1 = Mild injury with cytoplasmic vacuolation and focal nuclear pcnosis; Grade 2 = Moderate to severe injury with nuclear pcnosis, cytoplasmic hypereosinophilia and loss of intercellular borders; Grade 3 = Severe necrosis with disintegration of hepatic cords, hemorrhage and neutrophil infiltration [19].ii

### 2.10. Statistical analysis 

The data obtained after the study were evaluated statistically using the SPSS package program (IBM SPSS Statistics for Windows, 22.0. Armonk, NYVersion ). Mean, median, standard deviation and frequency values were used in the descriptive statistics of the data. Three groups were analyzed using the nonparametric Kruskal: IBM Corp.-Wallis test. Mann Whitney-U test was used for paired comparisons in subgroup analysis. A p value of <0.05 was considered significant.

## 3. Results

Serum ALT, AST, ALP, LDH, total and direct bilirubin levels were low in the control group while it was higher in the IR and BA+IR group (p<0.01). There was no statistically significant difference between these two groups. In terms of albumin values, albumin values ​​of the control group were higher, but there was no difference between the IR group and the BA+IR group (Table).

**Table:** Table. Laboratory findings of study groups [the results were presented as median and 95% CI (confidence interval)].

	a	b	c				
	GROUP I	GROUP II	GROUP III	P	P1	P2	P3
SCUBE1*(ng/mL)	12.27(10.5–14.88)	19.42(15.96 - 23.64)	17.17(13.63–22.10)	<0.01	<0.01	0.01	0.367
TOS**(μmol H2O2 equiv./L)	6.51(4.63–17.49)	29.48(20.77–43.95)	19.98(13.11–29.85)	<0.01	<0.01	0.02	0.104
TAS***(μmol Trolox equiv./L)	2.47(2.37–2.71)	2.28(2.12–2.67)	3.29(2.61–3.49)	<0.01	0.16	0.01	<0.01
OSI (TOS/TAS)#	3.69 (1.89–6.51)	12.99 (9.36–19.1)	6.43 (3.76–9.62)	<0.01	0.01	0.02	<0.01
IMA(absorbance unit)	0.875(0.824–0.900)	0.913(0.867–0.924)	0.920(0.874–0.952)	0.07	0.234	0.065	0.104
Albumin##(g/dL)	3.7(3.5–3.9)	2.75(2.68– 3.1)	2.85(2.52–3.1)	<0.01	<0.01	<0.01	0.995
IMAR (IMA/Albumin)###	0.234 (0.223–0.24)	0.325 (0.291–0.34)	0.323 (0.291–0.37)	<0.01	<0.01	<0.01	0.699
ALP+(U/L)	108(46.9–201.7)	271(210.5–393.7)	250(219.6–289.6)	<0.01	<0.01	<0.01	0.645
ALT+(U/L)	52(45–56.1)	141(96.3–191)	122(97.7–226.3)	<0.01	<0.01	<0.01	0.959
AST+(U/L)	96(72–133)	568(398–763)	545(405–763)	<0.01	<0.01	<0.01	0.999
TBIL+(mg/dL)	0.275(0.24–0.34)	10.08(2.464–12.19)	9.73(2.28–11.09)	<0.01	<0.01	<0.01	0.505
DBIL+(mg/dL)	0.01(0.008–0.02)	6.4(1.1–7.4)	5.8(0.991–7.1)	<0.01	<0.01	<0.01	0.507

NOTE = the values are presented as median (min-max). Values are given as median (95%CI). p – between three groups; p1 – between group-I and group-II; p2 – between group-I and group-III; p3 – between group-II and group III.

**Figure 1:**
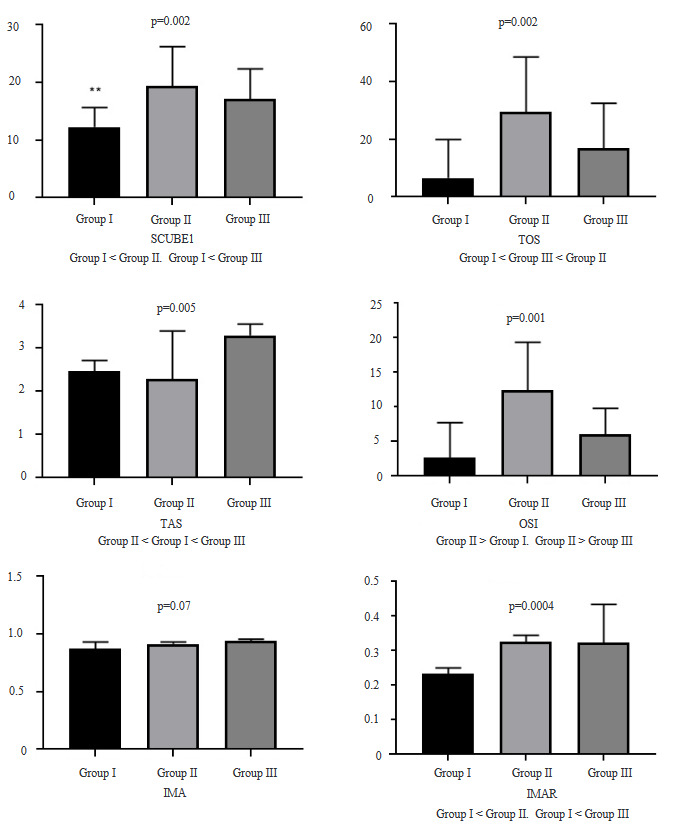
SCUBE-1, TOS, TAS, OSI, IMA, and IMAR levels between groups. SCUBE-1 levels of group I was significantly lower than the other groups (p < 0.05). TAS of the group III was found significantly higher (p = 0.02). TOS is significantly different from each other (p = 0.0019). IMA levels between 3 groups were compared no significant difference (p > 0.05).

For SCUBE-1 levels the control group was significantly lower than the other groups (p <0.05). The values ​​of the group given BA were lower between the IR group and the BA+IR group, this difference was not statistically significant. (p = 0.28) (Figure 1)When IMA levels between the 3 groups were compared no significant difference was found between the groups (p>0.05).

When we look at the total antioxidant capacity (TAS) the antioxidant capacity of the group given BA was significantly higher (p=0.02). When the total oxidant capacity (TOS) is examined all three groups were significantly different from each other (p

.=0.0019) While the control group had the lowest TOS values the IR group had the highest TOS values. While TOS values of the group given BA were significantly higher than the control group it was significantly lower than that of the IR group.

**Figure 2:**
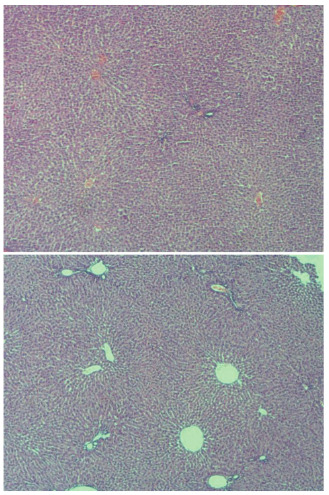
A,B. Normal histopathological appearance of the liver in the control group (H&E, X40) There was no or minimal hepatic damage in the livers of rats in the control group.

**Figure 3:**
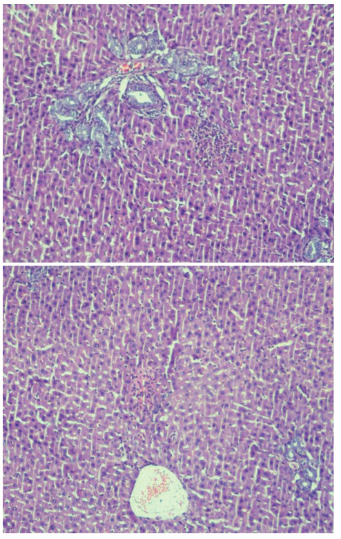
A, B: Intercellular border loss in hepatocytes, diffuse nuclear pycnosis, mild-moderate neutrophilic cell infiltration (grade 2) (H&E, X100). In the IR group loss of intercellular border loss in hepatocytes, diffuse nuclear pycnosis and mild to moderate neutrophilic cell infiltration were observed.

Hepatic damage degree of IR group was higher than other groups (p<0.001). There was no statistically significant difference between control group and BA+IR group for grade parameter (p=0.36). There was no or minimal hepatic damage in the livers of rats in the control group (Figure 2AB). In the IR group loss of intercellular border loss in hepatocytes, diffuse nuclear pycnosis, and mild to moderate neutrophilic cell infiltration (grade 2) were observed (Figures 3AB). Dissociation, hemorrhage and severe neutrophilic cell infiltration in hepatocytes in rats with IR (grade 3) were seen (Figure, 4 AB). Liver damage was minimal in rats given BA before IR (Figure 5AB),.,

**Figure 4:**
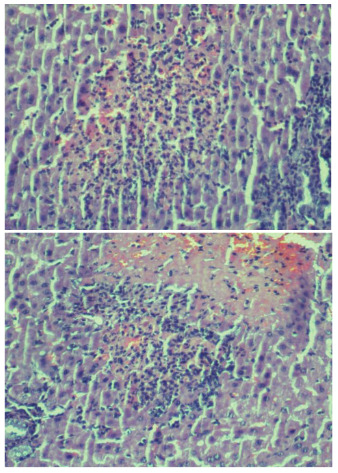
A, B: Structural disarrangement of liver parenchyma, hemorrhage, necrotic cells, and severe neutrophilic cell infiltration in liver of rats with ischemia reperfusion (grade 3) (H&E, x200). Dissociation, hemorrhage, and severe neutrophilic cell infiltration in hepatocytes in rats with IR were seen.

**Figure 5:**
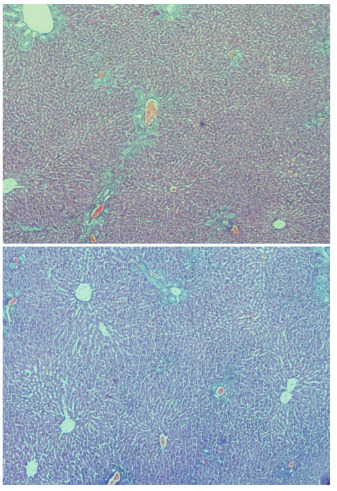
A, B: Minimal damage to the liver parenchyma of rats given boric acid (H&E, x40). Liver damage was minimal in group III. (Figures 5A and B).

## 4. Discussion

Liver IR injury is common in complicated surgical procedures, major trauma and transplantation cases. Although ways are sought to reduce this damage the development of IR injury cannot be prevented. It is emphasized that antioxidant substances should be given before ischemia develops. Experimental studies are still ongoing because an effective method has not been found yet. 

The most important finding in our study is that BA exerts a hepatoprotective effect on rats that we have experimentally developed IR injury of cholestatic liver. Both histopathological and biochemical results of this study showed better results in the BA given group compared to the control group in terms of liver function and tissue samples. 

The liver has a serious blood flow to maintain many synthesis and secretion functions. Therefore, ischemia is very sensitive to reperfusion. Damage during reperfusion is divided into early and late periods. While cellular damage is seen 26 after reperfusion in the early period neutrophil dominance is observed in the late period. While free oxygen radical-mediated damage is prominent in the early period neutrophil-mediated inflammatory response is dominant in the late period [20]. Another effective mechanism in IR injury is the impairment of intracellular Ca-hours ^2^^+^, Na^+^, H^+^ balances by decreasing mitochondrial ATP production. The intracellular increase of Ca^2^^+^ increase FOR production and triggers apoptotic mechanisms. Cholestasis is a clinical condition that occurs as a result of arrest or slowing of bile flow due to obstruction in intra or extrahepatic bile ducts. While cholestasis alone increases Kupffer cell activation and may cause FOR release. IR injury that develops in the presence of cholestasis is much more severe [21]. Many surgeons expect the pre-operative bilirubin levels to decrease in patients with very high bilirubin levels in elective operations. 

There are many studies in the literature regarding liver IR injury and its prevention. The model of liver IR injury in cholestatic rats is a newer experimental model. So there are fewer studies. This study was planned because BA has antioxidant effects and boron is a mineral found in our country.

Trace element boron which is the characteristic element of BA may have significant effects on various metabolic and physiological systems in organism [22]. The effects of BA on vitamin, enzyme, hormone, energy and mineral metabolism have been shown in some studies [11,23]. The researchers explain the biological effects of BA by two different hypotheses. In the first hypothesis it is argued that BA is negative regulator affecting the path of competitive inhibition in key enzyme reactions [24]. In the second hypothesis it is argued that BA plays an important role in cell membrane function, structure and stability [25]. 

In a study aimed to use BA as an antioxidant to protect liver from IR injury they found this beneficial effect probably originated from radical scavenging activity of BA [26]. In addition, BA pretreatment prevented ischemia induced alterations in liver tissue biomolecules. Supporting, the protective effect of BA against liver damage induced by ly carbon tetrachloride (CCl4) was found in mice [27]. esides, BA decreased inflammation, oxidative stress and apoptosis caused by cisplatin toxicity and increased ER stress in kidney [28]. B Sogut et al indicated antioxidant and antiapoptotic effects of boric acid on hepatoxicity in chronic alcohol fed rats [29]. In our study we found BA was seen to significantly reduce inflammation and fibrosis by examination of the liver tissue samples histopathologically. Besides according to our findings BA increased the antioxidant capacity and decreased the oxidant capacity when TAS /TOS levels were examined. On the other hand further studies need to focus on highlight the underlying these effects/mechanisms of BA protection.

 IMA is an albumin that changes its structure as a result of ischemia. In addition to ischemia IMA may increase in acute coronary syndrome, cerebrovascular diseases, liver failure, end-stage renal disease, severe traumas, pulmonary embolism, sepsis, diabetes, systemic sclerosis, some neoplastic diseases and infections [3032]. In a study conducted with IMA in chronic liver disease. individuals with chronic liver disease and 28 healthy individuals were compared. IMA values were found to be significantly higher but AST and ALT albumin values did not correlate with th high. IMA was inversely proportional to albumin [33]. In a study conducted on 33 healthy children and 33 children with chronic liver disease, a correlation was found between fibrous scoring and IMA and no relationship was found with other biochemical parameters [34]. In a study in which an experimental acute mesenteric ischemia model was applied-43 is although an increase was observed in IMA values at 0, 1, 3 and 6 no significant difference was found [35]. In our study albumin values of the control group were high while the albumin values of the IR group and the group given BA were low. There was no statistically significant difference between these two groups. Although there was a difference in albumin values for 3 groups when IMA levels were compared no statistically significant difference was found between 3 groups.

Although there are many methods for evaluating parenchymal damage in liver IR injury the most frequently used measurements are serum AST, ALT and LDH values [36]. When we looked at these values in our study we saw that the values of the group 1 were low while the IR group and BA group were high. When the IR group and the BA group were compared there was no statistically significant difference.

Cholestasis occurs in many situations in the clinic. The most common causes are gallstones and benign and malignant tumors. We followed the rats for week after ligation of the common bile duct to induce experimental obstructive jaundice.In the case of cholestasis serum bilirubin levels and ALP levels are the most investigated parameters. When the groups were compared total and direct bilirubin levels and ALP were lower in the control group while it was higher in the IR group and BA+IR group. There was no statistically significant difference between the IR and the BA+IR groups. The therapeutic effect of BA in the early period did not reflected to associated laboratory valueshours one for ALT/AST and bilirubin/ALP levels and as a result there is not a statistically significant difference was found between IR and IR-BA groups. 

SCUBE-1 is a new biomarker. Lindemann et al. found the SCUBE-1 cDNA fragment restricted in the 22q13 chromosome in the endothelium and showed a fibrin-rich region within the organized thrombus in platelets [37]. SCUBE-1 is stored in platelet α-granules and replaced by platelet stimulation and activation at the cell surface. Platelet SCUBE-1 exposed to the surface mediates agglutination under thrombotic conditions. Studies have reported in the literature that plasma SCUBE-1 values started to increase in 6 hours after platelet activation and can be measured in plasma for an average of 34 days. There are studies showing that SCUBE-1 levels increase in pulmonary thromboembolism [38]. SCUBE-1 levels have been shown to increase in experimental ischemic stroke [39,40]. Another study showed that SCUBE-1 levels started to increase within 2 in acute mesenteric ischemia [41]. One study reported that the SCUBE-1 level increased in hemodialysis patients with no ischemic symptoms and they attributed this to platelet dysfunction due to hemodialysis [42]. In a study evaluating patients with gastric cancer high SCUBE-1 values were found and it was suggested that this may be associated with thrombosis due to malignancy [43].

In our study, while SCUBE-1 levels were low in the IR group it was significantly higher in the IR and BA+IR group. This difference was not statistically significant in the BA+IR group although it was lower in the IR group. With these results, it is thought that SCUBE-1 can be used as a biomarker in liver IR injury. Further studies are needed on this subject.When we look at the total antioxidant capacity we see that the group given BA was significantly higher than the group 1 and IR groups. When we look at the total oxidant capacity we see that the 3 groups are significantly different from each other while the control group has the lowest oxidant capacity while the IR group has the highest oxidant capacity. BA group was statistically significantly lower than IR group. With these data we can say that BA increases antioxidant capacity and decreases oxidative stress.

Although many mechanisms play a role in the pathophysiology of IR injury the most important ones are the increase of FOR and the increase of intracellular calcium. While the increase in intracellular calcium increases FOR levels, intracellular calcium increases further with the disruption of the cell membrane structure. FOR causes lipid peroxidation in the cell and organ membranes [44]. It has been shown that boric acid reduces the levels of malondialdehyde (MDA) which is a lipid peroxidation product in aluminum-induced hepatotoxicity [45]. It has been shown that the levels of superoxide dismutase (SOD) enzyme which is an antioxidant in hepatotoxicity induced by carbon tetrachloride increase with BA [27].-hours 

As a result of the activation of macrophages with reperfusion, pro-inflammatory cytokine release increases in the environment. In the studies of Başbuğ et al. proinflammatory cytokines IL-6 and TNF-α were found to be higher in the hepatic IR group but decreased in the BA group [46].

Malondialdehyde (MDA) levels increased and antioxidant reduced glutathione (GSH). superoxide dismutase (SOD), and catalase (CAT) levels were found to be decreased in the gentamicin-induced oxidative stress experiment model in rats; in the group given BA it was shown that MDA levels decreased. GSH, SOD and CAT levels increased [47]. In the ovarian IR experimental the administration of 2-aminoethoxydiphenyl borate (2-APB) showed that the TAS level was higher and the TOS level was lower than the IR group [48]. In a study conducted. It was shown that BA increased GSH. SOD and CAT levels in formaldehyde-induced oxidative stress, decreased the level of MDA and decreased the pro-modelinflammatory level of TNF-α [49]. In the experimental necrotizing enterocolitis model it has been shown that BA increases antioxidant mechanisms and decreases pro-inflammatory IL-6 and TN-α [50].

2-aminoethoxydiphenylborate (2-ABP) one of the boron compounds is used in calcium channel stabilization. Increase in intracellular and mitochondrial calcium forms the basis of IR injury. In the literature, studies showing that IR damage can be reduced by inhibiting intracellular and microcrystalline calcium increase with 2-ABP in the hepatic IR model [51,52].imitation of our study is that although studies in large sample size and large animal models are needed to evaluate the effectiveness for the clinical application-

L 

In conclusion, to the best of our knowledge this is the first experimental study in literature to have examined the effect of BA on experimental IR injury of the cholestatic liver. Liver tissue samples were examined histopathologically and BA was seen to significantly reduce inflammation and fibrosis. Again when TAS/TOS levels were examined it was seen that BA increased the antioxidant capacity and decreased the oxidant capacity. Thus the results of the study demonstrated the hepatoprotective effect of intraperitoneal BA administration before IR injury of the cholestatic liver. Nevertheless, further experimental and clinical studies are needed to better understand the protective role and mechanism of BA. 
